# Current examining methods and mathematical models of horizontal transfer of antibiotic resistance genes in the environment

**DOI:** 10.3389/fmicb.2024.1371388

**Published:** 2024-04-04

**Authors:** Fan Liu, Yuqiu Luo, Tiansi Xu, Hai Lin, Yong Qiu, Bing Li

**Affiliations:** ^1^School of Energy and Environmental Engineering, University of Science and Technology Beijing, Beijing, China; ^2^School of Environment, Tsinghua University, Beijing, China

**Keywords:** antibiotic resistance genes, horizontal gene transfer, examining methods, mathematical models, transfer dynamics

## Abstract

The increasing prevalence of antibiotic resistance genes (ARGs) in the environment has garnered significant attention due to their health risk to human beings. Horizontal gene transfer (HGT) is considered as an important way for ARG dissemination. There are four general routes of HGT, including conjugation, transformation, transduction and vesiduction. Selection of appropriate examining methods is crucial for comprehensively understanding characteristics and mechanisms of different HGT ways. Moreover, combined with the results obtained from different experimental methods, mathematical models could be established and serve as a powerful tool for predicting ARG transfer dynamics and frequencies. However, current reviews of HGT for ARG spread mainly focus on its influencing factors and mechanisms, overlooking the important roles of examining methods and models. This review, therefore, delineated four pathways of HGT, summarized the strengths and limitations of current examining methods, and provided a comprehensive summing-up of mathematical models pertaining to three main HGT ways of conjugation, transformation and transduction. Finally, deficiencies in current studies were discussed, and proposed the future perspectives to better understand and assess the risks of ARG dissemination through HGT.

## 1 Introduction

Antibiotics represent one of the most significant inventions of the 20th century, playing a crucial role in safeguarding human life (Mac Lean and San Millan, 2019). However, the escalating use of antibiotics has led to the emergence and widespread dissemination of antibiotic resistant bacteria (ARB) and antibiotic resistance genes (ARGs). Various high-abundance ARGs have been detected in diverse environments such as soil, rivers, and feces, with ARB and ARGs even found in the environment devoid of antibiotic usage (Knapp et al., 2010; Di Cesare et al., 2012; Chi et al., 2020; Jang et al., 2022). Currently, the global annual death toll due to antibiotic resistance is estimated at a staggering 700,000, with the World Health Organization projecting a surge to 10 million deaths by 2050 (Pruden et al., 2006; Molnar, 2019). The emergence and dissemination of ARGs have introduced potential ecological and health risks.

The dissemination of antibiotic resistance involves two pathways, namely horizontal gene transfer (HGT) and vertical gene transfer (VGT). HGT is predominantly composed of plasmid-mediated conjugation, extracellular DNA-mediated transformation, phage-mediated transduction, and recently discovered vesiduction. HGT exhibits greater potential for ARG transmission compared to VGT, facilitating ARG transfer not only between different bacterial strains but also across distinct species (Andam et al., 2011; Zarei-Baygi and Smith, 2021). Undoubtedly, antibiotics can facilitate HGT, while it is also influenced by various non-antibiotic factors, constituting a current research hotspot (Warinner et al., 2014; Zhang et al., 2018; Jin et al., 2020). Presently, examining methods of ARG transfer predominantly encompass traditional culture method, microfluidics, and bioinformatics method, which the focus primarily centers on conjugative transfer. Each research methodology possesses its own merits and limitations. A systematic synthesis of these approaches is imperative at this juncture, fostering a comprehensive understanding that would enable subsequent researchers to judiciously select methodologies based on their specific research objectives.

The HGT dynamics is exceedingly intricate. In addition to examining methods, mathematical models furnish a simulated environment that can be informed by real-life data, serving as powerful tools for studying the dynamics of ARG transfer (Leclerc et al., 2019). However, the accuracy of mathematical models is contingent upon the quality and richness of the source data (Sorensen et al., 2005; Bakkeren et al., 2019). Consequently, the current trend involves combining experimental and simulation approaches. Mathematical models can be classified into “deterministic models” and “stochastic models,” with the former consistently producing the same results for a given set of parameter values, while the latter incorporates variability arising from random events in its outcomes (Tuljapurkar, 1991). Levin’s mass-action model, an early investigation into the dynamics of bacterial plasmids in homogeneous systems (Levin et al., 1979), provided insights into the conditions favoring the selection of mobile genetic elements (MGEs) in natural systems. Building upon this foundation, the introduction of spatially mathematical models addressed gaps in our understanding of plasmid dynamics in attached states (Fox et al., 2008; Connelly et al., 2011; Zhong et al., 2012). Currently, mathematical model development primarily focuses on conjugation dynamics, but failing to provide a comprehensive overview of the current plasmid dynamics model. Furthermore, there is a growing recognition of the research into transformation dynamics and transduction dynamics, but a systematic synthesis of such studies is still lacking.

The review initially delineated the general mechanisms of HGT, encompassing conjugation, transformation, transduction and vesiduction. Subsequently, the current examining methods of HGT were reviewed, including traditional culture, CoMiniGut, microfluidics, and bioinformatics method. The advantages, limitations and obtainable information were summarized. Next, a summary of existing mathematical models concerning the HGT dynamics, with a specific focus on the developmental status of conjugation models and a concise summation of limited transformation and transduction models. Finally, a critical analysis was conducted on the limitations of existing research, culminating in a forward-looking perspective on future research directions.

## 2 General routes of horizontal gene transfer

### 2.1 Conjugation

**Figure 1:**
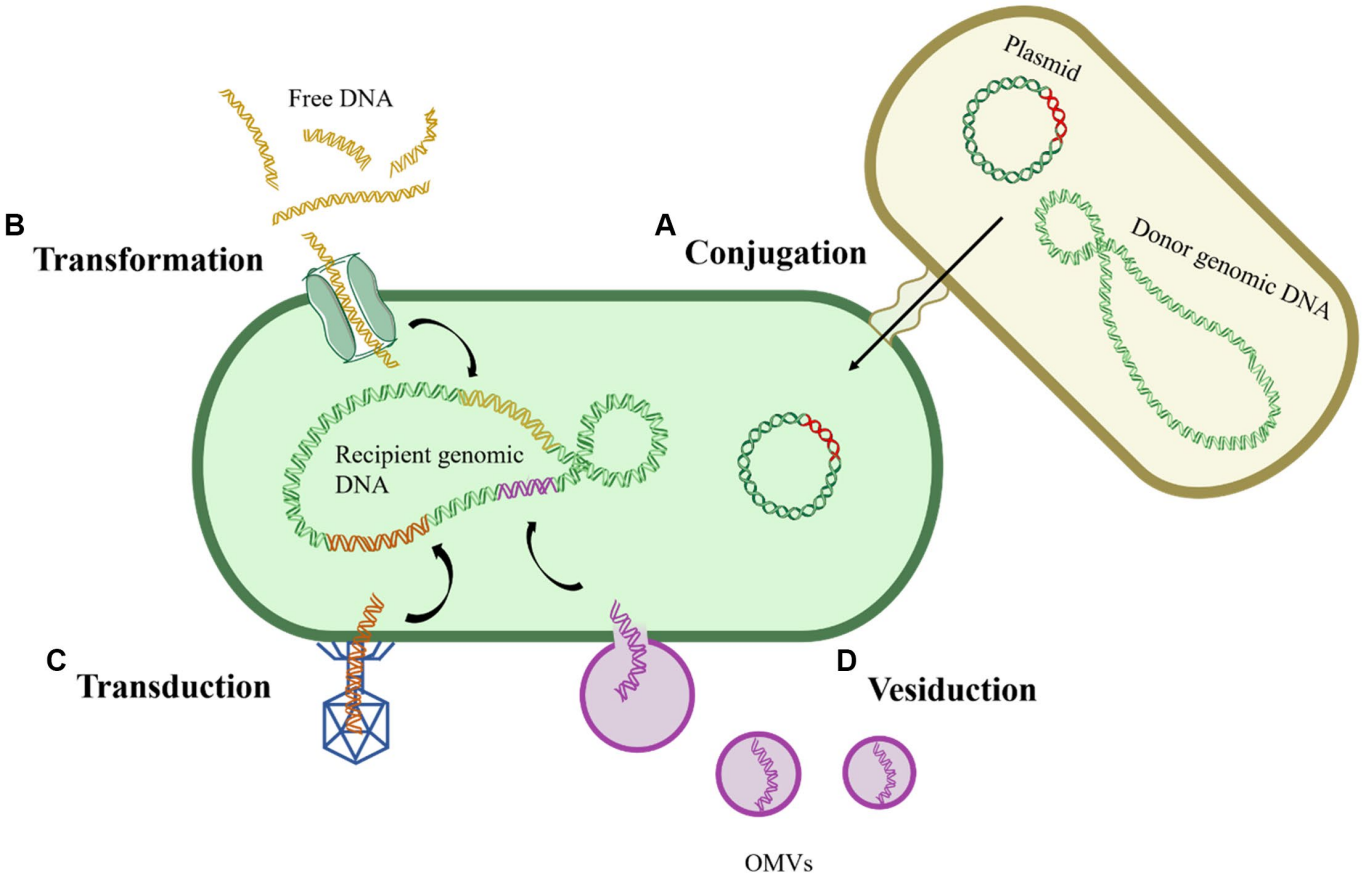
General routes of horizontal gene transfer. **(A)** Conjugation; **(B)** Transformation; **(C)** Transduction; **(D)** Vesiduction.

Conjugation refers to the process wherein donor and recipient bacteria, upon physical contact, establish a stable bridge through pili or channels, facilitating the transfer of MGEs, typically plasmids or transposons, from the donor bacteria to the recipient bacteria (Figure 1A). Conjugative plasmids, equipped with tra gene encoding the complete transfer enzyme, can spontaneously move from one cell to another, as exemplified by the F plasmid in *Escherichia coli* (*E. coli*), in the coexistence of non-conjugative plasmids and conjugative plasmids, both can undergo conjugative transfer (Davison, 1999; Qiu et al., 2012). The host range of plasmids is extensive, as conjugative transfer has been observed not only between bacteria of the same genus but also across different genera, and even between different biological species (Li and Zhang, 2022). Compared to bacterial suspension state, biofilms provide bacteria with more stable physical contact conditions, potentially enhancing the occurrence of conjugation. It was found that the conjugative transfer frequency of *Staphylococcus aureus* in biofilms was 10^4^ times higher than in suspended states (Sandberg et al., 2009). Among the various pathways of HGT, conjugation is considered the most significant and remains the most actively investigated route.

### 2.2 Transformation

Transformation refers to the process wherein bacteria directly uptake and integrate free DNA fragments from the extracellular environment, acquiring corresponding hereditary traits in the process (Magasanik, 1999) (Figure 1B). Unlike conjugation, transformation does not necessitate physical contact between donor and recipient bacteria; rather, it relies solely on the genetic encoding and regulation within the recipient bacterium (Johnsborg et al., 2007). The occurrence of transformation requires the simultaneous fulfillment of two conditions: the presence of free DNA fragments and competent cells, refer to those that have undergone alterations in cell membrane permeability under specific environmental pressures, with over 80 such species identified to date (Johnston et al., 2014; Liu et al., 2017). Given the widespread occurrence of transformation, researchers speculate the presence of potentially undiscovered bacteria with transformative potential in the environment.

### 2.3 Transduction

Transduction refers to the process by which phages erroneously package a portion of the host bacterium’s genes (frequency: 10^−5^–10^−7^) into their heads and transfer these genes to another cell through infection, thereby endowing the recipient cell with the corresponding hereditary traits (Li and Zhang, 2022) (Figure 1C). Transduction is classified into generalized transduction and specialized transduction, with the latter exclusively packaging bacterial DNA near the attachment site of the phage. In contrast, generalized transduction can randomly package any gene from the host into the phage head (Huddleston, 2014). However, due to the specificity of phages, transduction is not suitable for the horizontal transfer of widely distributed genes. Nevertheless, it is crucial to note that the contribution of transduction to HGT in natural environments should not be underestimated (Liu et al., 2020). Furthermore, phages carrying various ARGs have been detected in urban sewage, surface water, animals, and human samples, underscoring phages as potential reservoirs for ARGs (Colomer-Lluch et al., 2014; Quiros et al., 2014).

### 2.4 Vesiduction

Recently, a novel gene transfer mechanism mediated by outer membrane vesicles (OMVs) has been identified, termed vesiduction (Figure 1D). Rumbo et al. were the first to discover that OMVs can mediate the transfer of resistance genes, and the transfer occurs rapidly, within a three-hour timeframe (Rumbo et al., 2011). OMVs are double-membrane spherical nanostructures (50–500 nm) generated during bacterial growth (Toyofuku et al., 2019). Several studies have detected plasmids, chromosomal DNA fragments, and phage DNA fragments within OMVs, suggesting their role as carriers for gene transfer (Abe et al., 2020). OMVs can protect DNA from degradation by DNAases or other environmental factors, playing a crucial role in HGT (Liu et al., 2020). However, there are currently significant gaps in the understanding of the exact mechanisms and influencing factors of vesiduction, making it a focal point for future HGT research.

## 3 Examining methods of HGT and obtained information

In recent years, examining methodologies for HGT studies have been consistently innovated, from traditional culture method to bioinformatics method. These approaches provide robust supports for a comprehensive understanding of the influencing factors and transfer mechanisms of HGT. The methods for examining HGT and obtained information are summarized in Supplementary Table S1.

### 3.1 Traditional culture

#### 3.1.1 Flask/well plate

**Figure 2:**
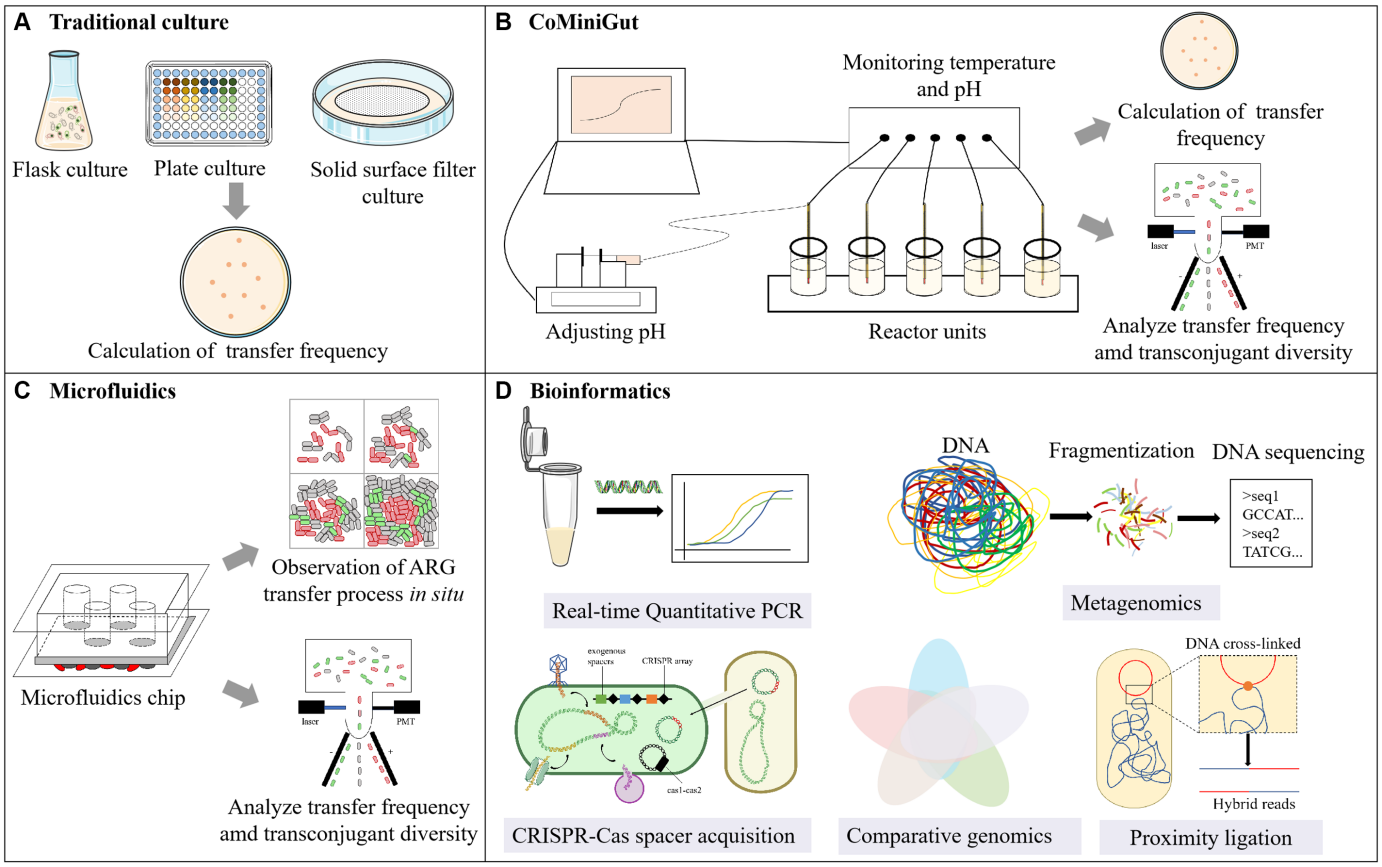
Examining methods of HGT. **(A)** Traditional culture; **(B)** CoMiniGut; **(C)** Microfluidics; **(D)** Bioinformatics.

Currently, our understanding of HGT is predominantly based on *in vitro* studies (Michaelis and Grohmann, 2023). The mating assay is commonly conducted using the flask/well plate culture method (Figure 2A), that involves mixing a set of donor cells (or gene fragments or phages) with recipient cells (or environmental samples), followed by culturing (McInnes et al., 2020). Therefore, it is applicable to investigations encompassing conjugation, transformation, transduction and vesiduction. Shaking flask or well plate cultivation is the most widely used in mating assays. In comparison with shaking flasks, well plate cultivation offers advantages such as small sample requirement, high-throughput analysis and parallelization, making it suitable for gradient concentration experiments.

Selective plate can be utilized to enumerate donor bacteria, recipient bacteria, and recipient bacteria acquiring new resistance in order to calculate the transfer frequency following mating experiments (Yuan et al., 2022; Zha et al., 2022). When utilizing fluorescently modified bacteria, the plasmid transfer frequency can be quantified through flow cytometry (Lin et al., 2023). However, when cultivating environmental samples, native microorganisms were often employed as recipient bacteria. Yet, the cultivability of native microorganisms is low (less than 1%), with the majority being in a non-culturable state (VBNC) and unable to form colonies on agar plates (Schottroff et al., 2018; Feng et al., 2021). This leads to biased results in cultivation-based detection, making this method more suitable for pure bacterial experiments. Additionally, selective agar plates can only calculate the transfer frequency at the endpoint of the experiment, unable to provide real-time information, and cultivation methods cannot distinguish between HGT and VGT (Li and Zhang, 2022).

#### 3.1.2 Solid surface filter

The major distinction between the membrane filter and the flask/well plate culture method lies in the solid nature of the culture medium, which is suitable for the study of conjugation. The donor and recipient bacteria were mixed on the membrane filter and cultured on the agar plate (Musovic et al., 2010; Klumper et al., 2017). Especially, in order to determining transfer frequency at the end of the assay, *in situ* visualization of transconjugants colonies are needed to emit fluorescence to calculated transfer frequency. In one previous study, the donor strain *E. coli* MG1655 utilized in their study chromosomally tagged with a gene cassette encoding constitutive red fluorescence protein (RFP) and carried a genetic tag encoding green fluorescent protein on the plasmid pKJK5 (Klumper et al., 2015). However, due to inhibitory effects, *E. coli* MG1655 exhibited only red fluorescence. The inhibitory effect disappears when this plasmid is transferred to non-fluorescent recipient bacteria, allowing for the expression of green fluorescent protein by the transconjugants.

Similar to flask cultivation, solid surface filter shares the characteristic of simplicity in operation. Nevertheless, filtration maximizes cell-to-cell contact, rendering it more suitable for simulating the conjugative transfer of ARGs on the surfaces of biofilms (Dechesne et al., 2005).

### 3.2 CoMiniGut

The gut has emerged as a focal point concerning the transfer of ARGs from exogenous bacterial species to indigenous microbiota. Accordingly, the *in vitro* gut model CoMiniGut has been developed for determining horizontal plasmid transfer under conditions that mimic the human colon environment, with a working volume of only 5 mL (Anjum et al., 2018; Wiese et al., 2018) (Figure 2). It comprises several parallel reactors units connected to a data logger. pH is regulated using injectors containing NaOH, while temperature control is maintained through the water bath device, ensuring stability within the simulated environment. Mehreen et al. utilized this system to regularly sample, with techniques such as the selective plate and Fluorescence-Activated Cell Sorting (FACS) to investigate the conjugative transfer frequency of *bla_CMY-2_* bearing plasmids in *E. coli*. Additionally, diversity analysis was conducted on the sorted transconjugants (Anjum et al., 2018, 2019).

Compared to other examining methods, CoMiniGut can simulate realistic intestinal environment, enabling the assessment of public health risks associated with the ARG transfer from exogenous *E. coli* (Anjum et al., 2018). Compared to animal models, CoMiniGut model exhibits high fidelity, offering more controlled experimental conditions such as temperature, pH, and oxygen concentration. Therefore, the establishment process of this system is relatively complex, necessitating extensive experimental validation to ensure its authenticity and accuracy, at a considerable cost as well.

### 3.3 Microfluidics

Building upon conventional cultivation methods, researchers have developed a method that combines cultivation and HGT detection simultaneously, utilizing a microfluidic chip. The microfluidic chip is a miniature experimental platform with microscale channels that can be used to control fluid flow. It can simulate both the physical and biological conditions relevant to microorganisms (Abe et al., 2020). Therefore, as an innovative tool, the microfluidic chip serves as a microreactor for microbial growth and mating assay, opening up new avenues for studying ARG transfer (Karimi et al., 2015; Li et al., 2018) (Figure 2C). Microfluidic chips equipped with delayed imaging, coupled with fluorescence technology, enable real-time observation of community changes and tracking of the transfer dynamics of ARGs, which is frequently employed in studies pertaining to conjugative transfer. In addition to the modified bacteria mentioned in Section 3.1.2, the process of infection and transduction caused by phages can be observed in real time by fluorescent labeling of phage (Doolittle et al., 1996; Tzipilevich et al., 2017), thus microfluidic chips also possess potential applications in the transduction research.

Microscopy enables *in situ* observation and single-cell imaging of ARG transfer, facilitating the differentiation between HGT and VGT. A microfluidic chip comprising eight parallel shallow micro-chambers was employed for distinguishing HGT and VGT. The chip was utilized for cultivating donor cells with plasmids encoding ARGs (RP4 and PKJK5) and recipient activated sludge bacteria. Plasmids were visualized *in situ*, and the transfer characteristics were analyzed (Li et al., 2019). In image analysis, the appearance of the first randomly occurring green fluorescence particle was considered as the initial sign of the first HGT. Based on this point, the extension of fluorescence particles due to cell growth and division was considered VGT, allowing for a clear distinction of the ratio between HGT and VGT during the diffusion of ARGs in biofilm structures. Additionally, another microfluidic chip containing a single channel was employed (Li et al., 2018; Qiu et al., 2018). In this chip, nutrients within the flow diffuse through the agarose membrane into the bacterial layer, concomitantly allowing the diffusion of metabolic waste out of the membrane, which is subsequently carried away by the flow. This chip facilitates the determination of dynamic features of the transfer process, encompassing cell growth rate, and kinetic variations in transfer frequency. This on-chip culture enables rapid exchange of substances and allows high density growth, and thus provides a close-to-*in vivo* model to study real-world biofilms. In a recent study, a microfluidic chip was employed featuring four identical cavities in lieu of channels, ensuring ample nutrient supply, evaluating the effect of heavy metal on ARG transfer between attached bacteria (Lin et al., 2019; Liu et al., 2022).

Furthermore, fluorescence combined with FACS enables high-throughput counting and screening of donors, recipients, and transconjugants, further 16S rRNA gene sequencing of the transconjugants can determine which host the plasmid has spread to (Klumper et al., 2015; Feng et al., 2021). However, this method requires artificial modification or labeling of bacteria, making it not only operationally complex but also prone to false positives or negatives. Additionally, fluorescence can only detect one plasmid in each experiment, and current research is mainly focused on *E. coli*, with other model strains yet to be developed.

### 3.4 Bioinformatics

Due to the natural environment is complex, the information obtained from laboratory settings are hardly to reflect the real conditions in the natural environment. Thanks to the evolving bioinformatics methods, we are gradually gaining insights into the HGT within natural microbial communities, allowing for a better characterization of the mechanisms underlying interactions between bacterial hosts and their genomes (Figure 2D). Moreover, the bioinformatics method is suitable for studying most HGT pathways.

#### 3.4.1 qPCR

Real-Time Quantitative PCR (RTQ-PCR), developed on this basis, utilizes fluorescence signal detection of PCR products. The intensity of the emitted fluorescence is proportional to the amount of amplified product, allowing for precise quantification of abundance and diversity of ARGs and MGEs (including plasmids, phages, insertion), assessing the occurrence and extent of HGT (Bartkova et al., 2021; Zheng et al., 2023). Wang et al. obtained relative gene copy numbers of 10 ARGs and MEGs through qPCR to predict the behavior of microbial communities (Wang et al., 2020). Keyes et al. ascertain the presence of resistance to Florfenicol in avian *E. coli*, an antibiotic not typically utilized in poultry (Keyes et al., 2000). qPCR is characterized by its sensitivity, accuracy, and independence from culture and expression. However, primer design for qPCR can be intricate.

#### 3.4.2 Sequencing technique

Metagenomics contributes to predicting HGT within microbial communities (Abe et al., 2020). It enables obtaining the distribution and occurrence of ARGs, bacteria, and MGEs in the environments without the need for cultivation, investigating the HGT processes through the distribution patterns and similarities of ARGs within microbial communities. Moreover, concurrent analysis of functional genes aids in elucidating the potential impact of the hosts carried ARGs on HGT (Wang et al., 2020). However, the data volume of metagenomics is substantial, usually provides relatively quantitative information with relatively low precision. Recent years, long-read sequencing technologies have been developed to obtain full-length MGEs such as plasmids and phages, facilitating the integration of host and microbial community genomic information, which contributes to a more comprehensive ecological perspective on HGT (Suzuki et al., 2019; Zeevi et al., 2019).

#### 3.4.3 Other novel methods

CRISPR-Cas spacer acquisition is a state-of-the-art method for real-time monitoring of HGT events at the nucleotide level (Munck et al., 2020). This process involves incorporating small foreign DNA fragments, or spacers, into CRISPR loci. Using an engineered “recording” strain with a plasmid housing the cas1-cas2 operon, mobile DNA entering the cell is captured and integrated into the CRISPR array (McGinn and Marraffini, 2019). This approach allows the study of transient HGT events, especially those with low transfer frequencies.

Comparative genomics allows for the prediction of MGEs based on contrasting the distribution of bacterial genomes closely associated with HGT (Smillie et al., 2011; Zhao et al., 2019). It can more accurately identify genes with similar sequences and functions; however, its sensitivity is contingent upon the reference genome employed. Proximity ligation techniques, such as Hi-C, facilitate the accurate linkage between MGEs and their hosts (Lieberman-Aiden et al., 2009). Consequently, tracking the host range of MGEs *in situ* and the origin of ARGs within environmental microbial communities is achievable, albeit with lower sensitivity and higher costs.

## 4 Mathematical models for HGT dynamics and frequency prediction

With the development of research methods for studying HGT in recent years, mathematical models serve as appropriate tools for further understanding HGT processes (Leclerc et al., 2019). The mathematical model primarily achieves two objectives. Firstly, mathematical models can be applied in the context of public health environments, that set against the backdrop of clinical medical environments are significant for designing effective antibiotic dosing regimens or developing drugs related to clinical infections (Roberts et al., 2021; Ali et al., 2022). Secondly, mathematical models set against real-world environments can assess and predict transfer dynamics under various influencing factors. For example, how the proportion of ARB population changed over time or under varying environmental conditions (Qu et al., 2016; Gothwal and Thatikonda, 2018). The fundamental characteristics and predictive content of mathematical models were summarized in Supplementary Table S2.

### 4.1 Conjugation models

The current research on HGT dynamics is predominantly focused on simulating conjugation process, encompassing two categories of model: deterministic models and stochastic models.

#### 4.1.1 Deterministic model

Models can be classified into deterministic models and stochastic models based on their types. Deterministic models yield fixed results for given parameter values and formulated as ordinary differential equations (ODE). The mass-action model proposed by Levin serves as the basis for many deterministic models and is applicable for estimating the ARG transfer frequency constant in fully mixed solutions (Levin et al., 1979). James et al. suggested that this model is equally applicable to studying plasmid transfer dynamics between donor and recipient bacteria in soil microcosms (Hall et al., 2016). The endpoint method proposed by Simonsen et al., based on Levin’s model, is commonly used to estimate plasmid transfer frequency constants on surfaces and in liquid-based cultures (Huisman et al., 2022). For instance, Laura et al. employed this method to investigate the plasmid dynamics of *E. faecalis* on biofilms, exploring the guiding role of quorum sensing systems in the conjugation process (Cook et al., 2011). However, many assumptions and simplifications in this classic method, such as ignoring the cost of plasmid carriage and assuming uniform growth rates for all strains, may lead to biases in estimating parameter consistency (Mishra et al., 2021). Therefore, researchers have examined the applicability of this model to conjugation dynamics in homogeneous liquid and biofilm environments, discovering that the model was effective for fully mixed planktonic environments, but maybe not suitable for predicting average behavior in biofilms (Shu et al., 2013).

The models applicable to simulating the conjugation dynamics of planktonic bacteria appear to be unsuitable for attached bacteria in nutrient-rich habitats, such as sewage plant fillers, plant rhizosphere surfaces, and soil treated with feces and so on (Fox et al., 2008). Therefore, there is a need to propose new models to complement the dynamics of bacterial plasmids in attached states. From this perspective, some studies have captured the fundamental characteristics of plasmid population dynamics in attached-state bacteria and proposed a spatially explicit mathematical model. Randal et al. substantiate the aforementioned perspective, demonstrating that, under nutrient-rich conditions, the density of *E. coli* carrying plasmid PB10 can increase from 10^−7^ to 13% (Fox et al., 2008). The model can also be applied to understand the conditions sustaining stable HGT behavior (Connelly et al., 2011).

Considering that some assumptions and simplifications in classical methods may lead to biased simulations, many researchers not only refer to the parameters of the original models but also combine simulations with experiments. The essential parameters were modified based on experiment conditions. For instance, Sulagna et al. considered plasmid carriage costs and external dependencies on the Levin mathematical model, making the conjugation model adaptable to variable conditions (Mishra et al., 2021). Additionally, microfluidic chip methods allow observations at the single-cell level, enabling *in situ* tracking of cell growth and plasmid transfer characteristics. This method provides significant assistance for future simulations of conjugation dynamics on biofilms (Li et al., 2014; Qiu et al., 2018).

#### 4.1.2 Stochastic model

Most stochastic conjugation models were agent-based, and the spatial distribution of populations was represented by discrete or continuous three-dimensional positions. In comparison to deterministic models, stochastic conjugation models can predict the dynamic behaviors of individuals (bacteria) or groups (population of microorganisms) and provide a new perspective for exploring the impact of spatial factors (Leclerc et al., 2019). As Supplementary Table S2 showed, Brian et al. used this approach to simulate a more realistic spatial environment to quantify conjugation frequency among ARB (Connelly et al., 2011). Zhong et al. developed a spatially explicit mathematical model that simulates the changing abundances of donors, recipients, and transconjugants over time, thereby calculating the conjugation frequency (Zhong et al., 2012). Artem et al. conducted a sensitivity analysis on steady-state changes induced by parameter variations (Novozhilov et al., 2005). Currently, the primary objective of stochastic models is to predict plasmid dynamics. For instance, Artem et al. investigated the evolution of horizontally transferred genes in microbial populations (Novozhilov et al., 2005), while Zhong et al. assessed the effectiveness of various plasmid transfer efficiency measures when they were applied to surface-associated populations (Zhong et al., 2012). However, development of stochastic models is relatively limited and needs further exploration and advancement.

### 4.2 Transformation models

Transformation models are gradually developed referring to the establishment process of conjugation models. Lu et al. adapted from the mass action law, allowing the transformation frequency by the proposed model to be influenced by varied DNA or cell concentrations (Lu et al., 2015). This was the first transformation dynamics model, successfully predicting the natural transformation frequencies of tetracycline resistance genes in both motile and non-motile strains of *Azotobacter vinelandii*. Asgher et al. extended the aforementioned model by incorporating additional process pathways, such as variations in nutrient concentration, alterations in the density of susceptible bacterial population and changes in the extracellular DNA density in response to the population of antibiotic resistant and susceptible bacteria, thereby enhancing the capacity to better capture the underlying dynamics (Ali et al., 2022).

*Acinetobacter baylyi* (*A. baylyi*) has become a commonly selected model bacterium in the transformation model-building process. Yue et al. based on the dynamics of *A. baylyi* ADP1, transformant populations, and the free plasmid pool, proposed and calibrated an ODE model to predict the transformation dynamics exposed to non-antibiotic drugs (Wang et al., 2022). Similarly, Yu et al. used the same model to predict the long-term effects of artificial sweeteners on transformation dynamics (Yu et al., 2022). Additionally, Robert et al. discovered that *A. baylyi* has a lytic effect on nearby *E. coli*, acquiring ARGs from neighboring bacteria considered as another way of transformation, based on this process, a population dynamic model with spatially structured microbial communities was established, quantifying transformation frequency on solid surfaces (Cooper et al., 2017).

### 4.3 Transduction models

There are two main types of transduction. Specialized transduction refers to the process in which the prophage erroneously excises DNA adjacent to the integration site (Chen et al., 2018). Generalized transduction involves the “erroneous packaging” of random bacterial DNA fragments, leading to the formation of transducing particles probably containing ARGs (Penades et al., 2015). Therefore, the limited objects of simulation regarding transduction currently focus on generalized transduction.

Volkova et al. initially proposed a mathematical model regarding the risk of antimicrobial resistance (AMR) spread through temperate phage transduction (Volkova et al., 2014). This study assumed a well-mixed and high-density cellular environment in the bovine intestinal tract, estimating the upper limit of transduction in *E. coli* due to generalized transduction (which is 10^−3^ times the same environmental conjugation rate). Moura et al. used individual-based models suggesting generalized transduction is a powerful mechanism of DNA transfer between strains, allowing the emergence of single and even double resistant variants (Fillol-Salom et al., 2019). The study proposed that the “erroneous packaging” of transduction is not accidental but an evolutionary characteristic of temperate phages in changing environments. Taking into account the no well-mixed host phage systems, Sankalp et al. developed a model with a small volume compartment to consider local effects (Arya et al., 2020). Through sensitivity analysis, it was discovered that transduction frequency was decreased in a more toxic environment, or with higher fitness costs of resistance or phage immunity.

Quentin et al. argued that the parameters of the three models mentioned above mainly rely on assumptions and previous studies, lacking reliable experimental data as evidence, which may limit the reliability of their results. Therefore, an interdisciplinary approach, combining experiments and simulations, was adopted to predict how phage-bacteria dynamics lead to the evolution of multidrug-resistant bacteria. The estimated transduction frequency was approximately 10^−8^ (Leclerc et al., 2023). Based on this, the model was further extended to simulate the impact of antibiotics on the phage-bacteria system under the same environmental conditions (Leclerc et al., 2022). The results indicated that the synergistic effect of phages and antibiotics rapidly killed bacteria but also led to faster ARG spread, depending on the interaction duration and antibiotics concentration. This simulation provides important guidance for future experimental and clinical work on the impact of phages on AMR evolution.

## 5 Future perspectives

The risks posed by the dissemination of ARGs to the environment and public health are non-negligible. Despite the ongoing expansion and refinement of studies for HGT, the examining methods, models and associated health risk assessment of HGT still need to be further explored. Future researches are proposed to address the following gaps:

1. Current research primarily focuses on conjugation and transformation, with a notable lack of investigation into transduction and vesiduction processes (Luo et al., 2023). Therefore, there is a necessity for a more comprehensive examination of the contributions of phages and OMVs to the dissemination of ARB in the environment, as well as the impacts of exogenous compounds (e.g., non-antibiotic drugs) on the induction of transduction and vesiduction, in order to better understand the contribution of HGT to ARG dissemination in the environment (Liu et al., 2020). Meanwhile, future researchers are urged to develop novel examining methods tailored to the objectives, such as *in situ* tracking and quantification of transduction and vesiduction and next-generation sequencing technologies with improved accuracy (Abe et al., 2020; Xiao et al., 2023).
2. Experimental and simulation approaches complement each other. Experiments can refine simulation parameters to establish more reliable models, while simulations can easily predict long-term trends that are challenging to obtain through experiments (Fox et al., 2008). Given the distinct mechanisms of different HGT pathways, there is a need for creative modeling studies on transformation, transduction and vesiduction of rather than simply extending existing conjugation models. Therefore, acquiring more experimental data is urgently needed to provide stronger support for the development of mathematical models (Abe et al., 2020). Additionally, incorporating environmental variables into existing models or utilizing experimental data from simulated real environmental systems, enabling simulated results to have higher practical significance (Leclerc et al., 2019).
3. Assessing public health risks associated with the dissemination of ARGs through HGT is of paramount importance (Sanganyado and Gwenzi, 2019). However, the majority of current research on HGT risk models primarily focus on foodborne infections, thereby overlooking the health risk HGT in the environment (Verraes et al., 2013). Hence, there is a need to integrate mathematical models with commonly used risk assessment models such as human exposure assessment model, causal model and dose–response model, etc. (Williams-Nguyen et al., 2016; Sanganyado and Gwenzi, 2019; Morgado-Gamero et al., 2021; Goh et al., 2022). The output of mathematical models will serve as inputs for risk assessment models, facilitating ongoing risk ranking and source tracking, thereby establishing an HGT risk assessment framework (Luo et al., 2023).

## 6 Conclusion

This review primarily delineates four general ways of HGT and then subsequently summarizes the advantages, limitations and obtainable information of current HGT examining methods. Following, combined with the experimental data, the development of existing HGT models was outlined. In the future, researches are proposed to focus more on transduction and vesiduction processes, efficiently utilize tools for examining HGT and develop mathematical models. Furthermore, combining HGT models with risk assessment models is also important direction for evaluation health risks of HGT in the environment.

## Author contributions

FL: Investigation, Writing – original draft. YL: Writing – review & editing. TX: Writing – review & editing. HL: Writing – review & editing, Project administration, Formal Analysis. YQ: Writing – review & editing, Funding acquisition. BL: Supervision, Writing – review & editing, Investigation, Funding acquisition.
