# Supplementary material for: Current examining methods and mathematical models of horizontal transfer of antibiotic resistance genes in the environment
Source: Front Microbiol. 2024 Apr 4;15:1371388. doi: 10.3389/fmicb.2024.1371388 (PMC11025395; doi:10.3389/fmicb.2024.1371388)
Supplement: Supplementary file 1 [file Table_1.docx]

Supplementary Material

# Supplementary Tables

# Table S1: Examining methods of HGT and obtained information

# Table S2: Mathematical models for HGT dynamics and frequency prediction

# Table S1. Examining methods of HGT and obtained information

| Examining methods | | Advantages | Limitations | Available information | Applied HGT pathways |
| --- | --- | --- | --- | --- | --- |
| Flask/well plate | Combined with selective plate culture | Simple operation;  High-throughput (well plate) | Not applicable to VBNC bacteria; Direct observation of bacteria and ARG transfer is not feasible; | Bacterial biomass; transfer frequency | Conjugation; Transformation; Transduction; Vesiduction |
| Solid surface filter | Combined with selective plate culture | Simple Operation; | Not applicable to VBNC bacteria; Direct observation of bacteria and ARG transfer is not feasible; | Transfer frequency | Conjugation |
|  | Combined with microscopy | In situ visualization; | Fluorescence modification of donor bacteria is required, not suitable for general bacteria | Transfer frequency |  |
| CoMiniGut | Combined with selective plate culture | Simulate authentic hut environments;  Controllable experimental conditions; Assess public health risks | Complex establishment process; High cost. | Transfer frequency | Conjugation |
|  | Combined with FACS |  |  | Transfer frequency; The diversity of transconjugants |  |
| Microfluidics | Combined with microscopy | In situ visualization; Simulate environmental conditions; Real-time monitoring | Fluorescence modification of donor bacteria is required, not suitable for general bacteria | Transfer dynamics and differentiation of HGT and VGT processes | Conjugation |
|  | Combined with FACS | High throughput; High sensitivity; Conducive to subsequent qPCR or sequencing analysis of transconjugants |  | Transconjugant analysis;  Transfer frequency |  |
| qPCR | / | Sensitivity; Accuracy; Independence from culture and expression | Primer design is intricate | Precise quantification of abundance and diversity of MGEs | Conjugation; Transformation; Transduction; Vesiduction |
| Sequencing technique | Metagenomics sequencing | Culture-Independent; Standardization; Comprehensive | Complex processing | Predicting HGT; Description of the distribution and occurrence of ARGs, bacteria, and MGEs; Linking MGEs to hosts |  |
|  | Long-read sequencing | Higher rate of capture than standard sequencing approaches | Potentially biased capture | Identifying mobile genetic element |  |
| Other novel methods | CRISPR-Cas spacer acquisition | Real time monitoring; Accurate | The system has presently only been developed for *few strains* | Ability to capture low-frequency HGT processes | Conjugation; Transformation; Transduction; |
|  | Comparative genomics | Accurate; Simple | The absence of an appropriate reference genome may lead to bias in the analysis results | Identifying mobile genetic element |  |
|  | Proximity ligation | Comprehensive | Low sensitivity, expensive | Linking MGEs to hosts |  |

**Table S2.** Mathematical models for HGT dynamics and frequency prediction

| Transfer mechanism | Type of model | Simulation environment/Influencing factors | | Bacteria | | Prediction content | Ref |
| --- | --- | --- | --- | --- | --- | --- | --- |
| Conjugation | Deterministic model | | Clinical medicine environment | | E. coli | Identify key strategies which would limit the emergence of antimicrobial-resistant bacterial strains | (D'Agata, Dupont-Rouzeyrol et al. 2008) |
|  |  |  | River water environment/fluoroquinolone, heavy metals | | Free/wild bacteria | The model was simulated for hypothetical pollution scenarios to predict the future conditions under various pollution management alternatives | (Gothwal and Thatikonda 2018) |
|  |  |  | Clinical medicine environment | | Superbugs with new type of resistance genes (NDM-1) | The dynamics of population in hospital environment where superbugs exist | (Qu, Pan et al. 2016) |
|  |  |  | Stored agricultural waste | | E. coli | Quantify the spread of antimicrobial resistance in stored agricultural waste | (Baker, Hobman et al. 2016) |
|  |  |  | Luria-Bertani (LB) medium/Natural organic matter (NOM) | | Two different *E. coli* strains | Prediction on RP4 plasmid conjugative transfer between bacteria under NOM exposure | (Li, Jiang et al. 2022) |
|  |  |  | A system composed of a large number of cells | | Pathogenic bacterial cells and immune cells | Bacterial growth, mutations, horizontal transfer and development of antibiotic resistance | (Knopoff and Sanchez Sanso 2017) |
|  |  |  | A single host animal (e.g. within the gut) | | E. coli | The development of AMR within a single host animal | (Roberts, Burgess et al. 2021) |
|  |  |  | LB medium | | E. coli | Facilitate meaningful comparisons of plasmid transfer frequencies in surface and liquid environments | (Zhong, Krol et al. 2010) |
|  |  |  | Natural environment | | Human pathogens | Construct a model framework for resistance emergence and used available quantitative data on relevant processes to identify limiting steps in the appearance of ARGs in human pathogens | (Bengtsson-Palme, Jonsson et al. 2021) |
|  |  |  | Clinical biofilm settings | | E. faecalis | A higher plasmid copy number in biofilm cells would enhance a switch-like behaviour in the pheromone response of donor cells with a delayed | (Cook, Chatterjee et al. 2011) |
|  |  |  | The scenarios encompassed: (i) large and small bacterial populations, (ii) strong and weak selection of the HGT events (transformants), and (iii) immediate or delayed sampling. | | Bacteria in natural populations | HGT of exogenous DNA into bacteria, the stochastic timing of rare HGT events is accounted for | (Townsend, Bohn et al. 2012) |
|  |  |  | Wastewater conditions/ Rifampicin | | Resistant populations in wastewater | The growth of resistant populations in different environmental scenarios | (Sutradhar, Ching et al. 2021) |
|  |  |  | A spatially structured, nutrient-rich environment | | E. coli | Plasmid dynamics | (Fox, Zhong et al. 2008) |
|  |  |  | LB medium/Prophage λ | | E. coli | Prophages can substantially limit the spread of conjugative plasmids | (Igler, Schwyter et al. 2022) |
|  |  |  | Luria broth/Phage | | E. coli | Quantify the infection kinetics of the nonlytic phage M13 and its impact on conjugation in the absence of selection pressure | (Wan and Goddard 2012) |
|  |  |  | LB medium | | E. coli | Calculate growth and plasmid transfer taking into account the fitness cost associated with plasmid carriage and temperature dependencies in vertical and horizontal gene transfer processes | (Mishra, Kluemper et al. 2021) |
|  |  |  | Soil microcosm | | P. fluorescens and P. putida | The evolutionary importance of alternative hosts to plasmid population dynamics in an ecologically relevant environment | (Hall, Wood et al. 2016) |
|  |  |  | Simulated gastric fluid | | E. coli | Estimate *E. coli* survival in gastric pH conditions as well as gene transfer from resistant to susceptibl in humans | (Hwang, Kim et al. 2017) |
|  |  |  | Water-sediment system | | Bacterial community in a  water-sediment ecosystem | The evolution of ARGs was mainly due to the direct effect of the change in bacterial community and HGT via the class 1 integron-integrase gene (*intI1*) | (Deng, Liu et al. 2020) |
|  |  |  | Animal gut | | E. coli | The dynamics of ceftiofur-sensitive and resistant commensal enteric Escherichia coli in the absence of and during parenteral therapy with ceftiofur | (Volkova, Lanzas et al. 2012) |
|  |  |  | LB medium | | E. coli | The conjugation dynamics of IncI1 plasmids carrying the blaCTX-M-1 gene in a batch culture and its impact on the population dynamics of three E. coli populations: donors, recipients and transconjugants | (Fischer, Dierikx et al. 2014) |
|  |  |  | Phosphate-buffered  saline (PBS) | | E. coli | Quantitatively describe the conjugation process and used this model to evaluate the effects of nano-TiO2 on the of ARGs | (Qiu, Shen et al. 2015) |
|  |  |  | M9 medium/Antibiotic | | E. coli | Quantifying conjugation dynamics in the presence and absence of antibiotic-mediated selection | (Lopatkin, Huang et al. 2016) |
|  |  |  | LB medium | | P. aeruginosa | Non-transmissible plasmids may experience episodes of horizontal gene transfer occurring at very low frequencies, and that these scattered transmission events are sufficient to stabilize these plasmids | (Pena-Miller, Rodriguez-Gonzalez et al. 2015) |
|  |  |  | LB or glucose-limited salt medium | | E. coli | Determining the rate parameter of conjugative plasmid transfer | (Simonsen, Gordon et al. 1990) |
|  | Stochastic model | | Natural environment | | Microbial populations | Evolution of horizontally transferred genes in microbial populations | (Novozhilov, Karev et al. 2005) |
|  |  |  | Surface environment | | E. coli | Evaluate the effectiveness of these various plasmid transfer efficiency measures when they are applied to surface-associated populations | (Zhong, Droesch et al. 2012) |
|  |  |  | Natural environment | | Microbial populations | Examine how conjugation can be maintained in populations; How both the costs of transfer and the benefits conferred affect evolutionary outcomes; How rates of transmission evolve, allowing this system to adapt to different environments. | (Connelly, Zaman et al. 2011) |
|  | Both | | Natural environment | | An asexually reproducing population of unicellular, prokaryotic organisms. | The influence that conjugation-mediated HGT has on the mutation-selection balance in an asexually reproducing population of unicellular, prokaryotic organisms | (Raz and Tannenbaum 2010) |
|  |  |  | Planktonic Environment and biofilm circumstances | | Enterococcus faecalis | The induction of conjugation in planktonic and biofilm circumstances | (Merkey, Lardon et al. 2011) |
| Transformation | Deterministic model | | LB medium/Artificial sweetener | | A. baylyi ADP1 | Predict the long-term effects on transformation dynamics under exposure to these sweeteners | (Yu, Wang et al. 2022) |
|  |  |  | Water environment | | Azotobacter vinelandii | The experimentally determined rates of natural transformation of a tetracycline resistance gene for motile and non-motile strains of *Azotobacter vinelandii* | (Lu, Massoudieh et al. 2015) |
|  |  |  | Clinical medicine environment | | Susceptible bacterial populations | Determine the best antibiotic dosing strategy | (Ali, Imran et al. 2022) |
|  |  |  | PBS solution | | A. baylyi | Predict the dynamics of transformation during exposure to non-antibiotic pharmaceuticals | (Wang, Lu et al. 2020) |
|  |  |  | Wastewater treatment plants (WWTPs) | | Sensitive bacterial community | The fate and transport of ARGs in receiving waters downstream of wastewater treatment plants | (Ikuma and Rehmann 2020) |
|  |  |  | Solid surfaces | | Acinetobacter baumannii | Quantifying killing and HGT on solid surfaces | (Cooper, Tsimring et al. 2017) |
| Transduction | Deterministic model | | Colibacillosisin calf | | E. coli | The infection dynamics ofenteric coliphages in commensal *Escherichia coli* in the large intestine of cattle | (Volkova, Lu et al. 2014) |
|  |  |  | Brain heart infusion broth (BHIB) | | Staphylococcus aureus | Phage-bacteria dynamics | (Leclerc, Wildfire et al. 2022) |
|  |  |  | Tryptic Soy Broth (TSB) | | S. enterica Typhimurium | Antibiotic susceptible cells become resistant to both antibiotics and phage by integrating the generalized transducing temperate phages and acquiring transducing phage particles carrying antibiotic resistance genes obtained from resistant cells in the environment | (Fillol-Salom, Alsaadi et al. 2019) |
|  |  |  | A scenario with a predominant antimicrobial sensitive population | | A single resistant cell | Transfer of resistance by transduction by lytic phages | (Arya, Todman et al. 2020) |
|  |  |  | BHIB | | Staphylococcus aureus | Identify conditions where phage and antibiotics act in synergy to remove bacteria or drive AMR evolution | (Leclerc, Lindsay et al. 2022) |

# Reference

Ali, A., M. Imran, S. Sial and A. Khan (2022). "Effective antibiotic dosing in the presence of resistant strains." *Plos One* **17**(10).

Arya, S., H. Todman, M. Baker, S. Hooton, A. Millard, J.-U. Kreft, et al. (2020). "A generalised model for generalised transduction: the importance of co-evolution and stochasticity in phage mediated antimicrobial resistance transfer." *Fems Microbiology Ecology* **96**(7).

Baker, M., J. L. Hobman, C. E. R. Dodd, S. J. Ramsden and D. J. Stekel (2016). "Mathematical modelling of antimicrobial resistance in agricultural waste highlights importance of gene transfer rate." *Fems Microbiology Ecology* **92**(4).

Bengtsson-Palme, J., V. Jonsson and S. Hess (2021). "What Is the Role of the Environment in the Emergence of Novel Antibiotic Resistance Genes? A Modeling Approach." *Environmental Science & Technology* **55**(23): 15734-15743.

Connelly, B. D., L. Zaman, P. K. McKinley and C. Ofria (2011). Modeling the Evolutionary Dynamics of Plasmids in Spatial Populations. 13th Annual Genetic and Evolutionary Computation Conference (GECCO), Dublin, IRELAND.

Cook, L., A. Chatterjee, A. Barnes, J. Yarwood, W.-S. Hu and G. Dunny (2011). "Biofilm growth alters regulation of conjugation by a bacterial pheromone." *Molecular Microbiology* **81**(6): 1499-1510.

Cooper, R. M., L. Tsimring and J. Hasty (2017). "Inter-species population dynamics enhance microbial horizontal gene transfer and spread of antibiotic resistance." *Elife* **6**.

D'Agata, E. M. C., M. Dupont-Rouzeyrol, P. Magal, D. Olivier and S. Ruan (2008). "The Impact of Different Antibiotic Regimens on the Emergence of Antimicrobial-Resistant Bacteria." *Plos One* **3**(12).

Deng, C., X. Liu, L. Li, J. Shi, W. Guo and J. Xue (2020). "Temporal dynamics of antibiotic resistant genes and their association with the bacterial community in a water-sediment mesocosm under selection by 14 antibiotics." *Environment International* **137**.

Fillol-Salom, A., A. Alsaadi, J. A. M. de Sousa, L. Zhong, K. R. Foster, E. P. C. Rocha, et al. (2019). "Bacteriophages benefit from generalized transduction." *Plos Pathogens* **15**(7).

Fischer, E. A. J., C. M. Dierikx, A. van Essen-Zandbergen, H. J. W. van Roermund, D. J. Mevius, A. Stegeman, et al. (2014). "The Incl1 plasmid carrying the bla<sub>CTX-M-1</sub> gene persists in <i>in vitro</i> culture of a <i>Escherichia coli</i> strain from broilers." *Bmc Microbiology* **14**.

Fox, R. E., X. Zhong, S. M. Krone and E. M. Top (2008). "Spatial structure and nutrients promote invasion of IncP-1 plasmids in bacterial populations." *Isme Journal* **2**(10): 1024-1039.

Gothwal, R. and S. Thatikonda (2018). "Mathematical model for the transport of fluoroquinolone and its resistant bacteria in aquatic environment." *Environmental Science and Pollution Research* **25**(21): 20439-20452.

Hall, J. P. J., A. J. Wood, E. Harrison and M. A. Brockhurst (2016). "Source-sink plasmid transfer dynamics maintain gene mobility in soil bacterial communities." *Proceedings of the National Academy of Sciences of the United States of America* **113**(29): 8260-8265.

Hwang, D., S. M. Kim and H. J. Kim (2017). "Modelling of tetracycline resistance gene transfer by commensal <i>Escherichia coli</i> food isolates that survived in gastric fluid conditions." *International Journal of Antimicrobial Agents* **49**(1): 81-87.

Igler, C., L. Schwyter, D. Gehrig and C. C. Wendling (2022). "Conjugative plasmid transfer is limited by prophages but can be overcome by high conjugation rates." *Philosophical Transactions of the Royal Society B-Biological Sciences* **377**(1842).

Ikuma, K. and C. R. Rehmann (2020). "Importance of Extracellular DNA in the Fate and Transport of Antibiotic Resistance Genes Downstream of a Wastewater Treatment Plant." *Environmental Engineering Science* **37**(2): 164-168.

Knopoff, D. A. and J. M. Sanchez Sanso (2017). "A kinetic model for horizontal transfer and bacterial antibiotic resistance." *International Journal of Biomathematics* **10**(4).

Leclerc, Q. J., J. A. Lindsay and G. M. Knight (2022). "Modelling the synergistic effect of bacteriophage and antibiotics on bacteria: Killers and drivers of resistance evolution." *Plos Computational Biology* **18**(11).

Leclerc, Q. J., J. Wildfire, A. Gupta, J. A. Lindsay and G. M. Knight (2022). "Growth-Dependent Predation and Generalized Transduction of Antimicrobial Resistance by Bacteriophage." *Msystems* **7**(2).

Li, H., E. Jiang, Y. Wang, R. Zhong, J. Zhou, T. Wang, et al. (2022). "Natural organic matters promoted conjugative transfer of antibiotic resistance genes: Underlying mechanisms and model prediction." *Environment International* **170**.

Lopatkin, A. J., S. Huang, R. P. Smith, J. K. Srimani, T. A. Sysoeva, S. Bewick, et al. (2016). "Antibiotics as a selective driver for conjugation dynamics." *Nature Microbiology* **1**(6).

Lu, N., A. Massoudieh, X. Liang, T. Kamai, J. L. Zilles, T. H. Nguyen, et al. (2015). "A kinetic model of gene transfer via natural transformation of Azotobacter vinelandii." *Environmental Science-Water Research & Technology* **1**(3): 363-374.

Merkey, B. V., L. A. Lardon, J. M. Seoane, J.-U. Kreft and B. F. Smets (2011). "Growth dependence of conjugation explains limited plasmid invasion in biofilms: an individual-based modelling study." *Environmental Microbiology* **13**(9): 2435-2452.

Mishra, S., U. Kluemper, V. Voolaid, T. U. Berendonk and D. Kneis (2021). "Simultaneous estimation of parameters governing the vertical and horizontal transfer of antibiotic resistance genes." *Science of the Total Environment* **798**.

Novozhilov, A. S., G. P. Karev and E. V. Koonin (2005). "Mathematical modeling of evolution of horizontally transferred genes." *Molecular Biology and Evolution* **22**(8): 1721-1732.

Pena-Miller, R., R. Rodriguez-Gonzalez, R. C. MacLean and A. San Millan (2015). "Evaluating the effect of horizontal transmission on the stability of plasmids under different selection regimes." *Mobile genetic elements* **5**(3): 1-5.

Qiu, Z., Z. Shen, D. Qian, M. Jin, D. Yang, J. Wang, et al. (2015). "Effects of nano-TiO<sub>2</sub> on antibiotic resistance transfer mediated by RP4 plasmid." *Nanotoxicology* **9**(7): 895-904.

Qu, L., Q. Pan, X. Gao and M. He (2016). "Population Dynamics of Patients with Bacterial Resistance in Hospital Environment." *Computational and Mathematical Methods in Medicine* **2016**.

Raz, Y. and E. Tannenbaum (2010). "The Influence of Horizontal Gene Transfer on the Mean Fitness of Unicellular Populations in Static Environments." *Genetics* **185**(1): 327-337.

Roberts, M. G., S. Burgess, L. J. Toombs-Ruane, J. Benschop, J. C. Marshall and N. P. French (2021). "Combining mutation and horizontal gene transfer in a within-host model of antibiotic resistance." *Mathematical Biosciences* **339**.

Simonsen, L., D. M. Gordon, F. M. Stewart and B. R. Levin (1990). "Estimating the rate of plasmid transfer: an end-point method." *Journal of general microbiology* **136**(11): 2319-2325.

Sutradhar, I., C. Ching, D. Desai, M. Suprenant, E. Briars, Z. Heins, et al. (2021). "Computational Model To Quantify the Growth of Antibiotic-Resistant Bacteria in Wastewater." *Msystems* **6**(3).

Townsend, J. P., T. Bohn and K. M. Nielsen (2012). "Assessing the probability of detection of horizontal gene transfer events in bacterial populations." *Frontiers in Microbiology* **3**.

Volkova, V. V., C. Lanzas, Z. Lu and Y. T. Groehn (2012). "Mathematical Model of Plasmid-Mediated Resistance to Ceftiofur in Commensal Enteric Escherichia coli of Cattle." *Plos One* **7**(5).

Volkova, V. V., Z. Lu, T. Besser and Y. T. Groehn (2014). "Modeling the Infection Dynamics of Bacteriophages in Enteric <i>Escherichia coli</i>: Estimating the Contribution of Transduction to Antimicrobial Gene Spread." *Applied and Environmental Microbiology* **80**(14): 4350-4362.

Wan, Z. and N. L. Goddard (2012). "Competition Between Conjugation and M13 Phage Infection in <i>Escherichia coli</i> in the Absence of Selection Pressure: A Kinetic Study." *G3-Genes Genomes Genetics* **2**(10): 1137-1144.

Wang, Y., J. Lu, J. Engelstadter, S. Zhang, P. Ding, L. Mao, et al. (2020). "Non-antibiotic pharmaceuticals enhance the transmission of exogenous antibiotic resistance genes through bacterial transformation." *Isme Journal* **14**(8): 2179-2196.

Yu, Z., Y. Wang, I. R. Henderson and J. Guo (2022). "Artificial sweeteners stimulate horizontal transfer of extracellular antibiotic resistance genes through natural transformation." *Isme Journal* **16**(2): 543-554.

Zhong, X., J. Droesch, R. Fox, E. M. Top and S. M. Krone (2012). "On the meaning and estimation of plasmid transfer rates for surface-associated and well-mixed bacterial populations." *Journal of Theoretical Biology* **294**: 144-152.

Zhong, X., J. E. Krol, E. M. Top and S. M. Krone (2010). "Accounting for mating pair formation in plasmid population dynamics." *Journal of Theoretical Biology* **262**(4): 711-719.
